# Criminal behavioral data analysis for recidivation estimation in convicted offenders

**DOI:** 10.1016/j.dib.2022.108323

**Published:** 2022-05-29

**Authors:** Aman Singh, Subrajeet Mohapatra, Madhumita Bhattacharya

**Affiliations:** a Department of Computer Science Engineering, Birla Institute of Technology Mesra, Ranchi-835215; b Department of Clinical Psychology, Central Institute of Psychiatry, Kanke Ranchi

**Keywords:** First-time Offenders, Recidivism, Data analysis, Behavior Analysis, Psychological factor

## Abstract

The act of continuing to commit crimes after being imprisoned for a first-time offence and freed is known as recidivism. The level of delinquent behavior in an individual character, that is closely associated to repeated recidivation, can be determined by assessing offenders behavioral features. The dataset includes 220 offenders, with a total of 204 participants whose data was used to create the desired dataset. The raw information was acquired using a questionnaire form that included personality traits, parental and family characteristics, socio-demographic characteristics, crime details, cumulative jail behavior elements, and the HCR-20 risk assessment technique. Behavior sample was gathered from several jails and correction facility in the Indian state of Jharkhand for the objective for initial relapse estimation from first convicts in the current study. The dataset can be used by criminologists, sociologists, psychologists, and academicians to determine an offender's pattern and psychological qualities. Specialists in detention centre undertook the felony evaluation.

## Specifications Table

**Table:** 

Subject	[Psychology, Computer Science]
Specific subject area	Criminology, Artificial Intelligence and Data Science]
Type of data	Tables
How the data were acquired	Survey* among convicted offenders Instruments: HCR-20, Semi-structure performa
Data format	Raw and Analysed (Excel)
Description of data collection	Data was collected of offenders who were convicted once or more aged between (18-45) years. A quantitative approach along with an standard risk assessment tool HCR-20 was employed among several convicted offenders for different crimes.
Data source location	Birla Institute of Technology, Mesra, Ranchi-835215
Data accessibility	Repository: “First time Offender Data”, Mendeley Data, V3, Data Identification Number- doi: 10.17632/8j3tf5zfd9.3 Link: http://dx.doi.org/10.17632/8j3tf5zfd9.3
Related research article	A. Singh and S. Mohapatra, ”Development of Risk Assessment Framework for First Time Offenders Using Ensemble Learning,” in IEEE Access, vol. 9, pp. 135024-135033, 2021, doi: 10.1109/ACCESS.2021.3116205.

## Value of the Data

• The dataset can be used by criminologists, sociologists, psychologists, and academicians to determine an offender's pattern and psychological qualities.
• Policymakers, probation officers, and the criminal justice system can utilise the data to make improved judgement on felony convictions, parole, bail, probation, and penal facilities for first-time offenders.
• The data can be utilised to spot high-risk criminals, repeat offenders with aggressive views, and people with anti-social personalities. As a result, the crime prevention programme can be enhanced.
• The information can be utilised to improve vocational and skill training to meet the needs of offenders.
• The information can be used to track down criminals, analyse their risks, and predict recidivism.
• The dataset can also be used to lower crime rates by providing proper rehabilitation, identify crime patterns, and determine how offenders can reintegrate back into society.

## 1 Data Description

First time offenders are those who got convicted for any illicit or unlawful act under Indian Penal Code for any crime. This article was based on a study of 220 criminals from the Indian state of Jharkhand [1] who had been convicted at least once in the previous five years. The dataset contains 220 offenders, with a total of 204 participants for the final dataset, as shown in the Table 1**.**

**Table 1:** Behavior data for each factor included in the questionnaire.

Factors	Attributes		Score/Range	No. of participants Identified
Demographic	Marital Status	Married	0	20
		Unmarried	1	184
	Qualification	Illiterate	2	128
		High School	1	44
		Intermediate	0	20
		Graduation and above	0	12
	Employment	Employed	0	116
		Unemployed	1	88
	Age		18-30	204

Socio-Economic	Health	Good	0	
		Bad	1	
	Financial status	Below BPL	1	130
		Above BPL	0	74

Family/Parental	Parent Conflict		2	Semi-structure Performa prepared by Panel
	Single/ Divorced		1	
	Parental Criminal Activity		2	
	Family financial condition		1	
	Parent education		1	
	Sibling relation		1	
	Sibling influence		2	
	Dysfunctional family		2	

Environmental	Friend Criminal		1	Semi-structure Performa prepared by Panel
	Friend's background		0	
	Greedy friends		1	
	Friend's family involvement in Crime		1	
	Neighbourhood and society influence		1	

In the survey questionnaire the data were filled by the clinical Psychologists, verified and authenticated by the panel of psychologists listed in Table 2 below.

**Table 2:** Expertise Panel and Experience details.

Sl. No.	Expertise Field	Experience in Years
1.	Psycho-Diagnostics and Behavior Therapy	15+ Years
2.	Child and Adolescent Psychiatry and Forensic Science	12+ Years
3.	Neuropsychology, Cognitive Behavior	5+ Years
4.	Bi-polar Disorder, Adolescent Behavior, Rehabilitation	8+ Years
5.	LLB, LLM, Criminology, Criminal Justice	20+ Years
6.	Pattern Recognition, Cognitive Modelling and Advance ML	10+ Years

The survey questionnaire consists of different behavior factors of an individual offender such as personality, parental and family, environmental, Demographic, Socio-economic, Offence details, a standard risk assessment tool HCR-20 [2] and cumulative prison behavior factors. The prison behavior, crime details and frequency of each participant were also collected and tabulated in Table 3.

**Table 3:** Crime, Incarceration and Prison Behavior details.

Sl. No.	Variables	Frequency	Percentage
1.	Type of Crimes		
	Murder and Conspiracy	52	25
	Sexual abuse and Abduction	96	47
	Theft or Fraud	22	10.7
	Burglary	16	7.84
	Physical violence	10	4.90
	Others	08	4

2.	Incarceration Duration		
	0-2 years	48	23.52
	2-5 years	104	50.90
	Above 5 years	52	25.48
3.	Prison Behavior and Participation		
	Willingness Mainstream	132	-
	Loneliness or Depression	120	-
	Participation in Skill training	Yes- 140 No- 64	-
	Psychological condition	Good- 124 Poor- 80	-

## 2 Experimental Design, Materials and Methods

### 2.1 Experimental design

The experimental set up consists of data verification and validation, data quality and data or features exclusion. A quantitative approach [3] based on semi-structure Performa survey questionnaire was employed to collect data among first time offenders who were convicted once for any criminal act under Indian penal Code. The questionnaire was developed based on the previous researches and were verified and validated by the expertise panel. The expertise panel consists of six members who have relevant experience with respect to the study area. The panel includes academicians, researches, criminal lawyer and subject expert with at least five years of experience in the relevant field.

The panel suggestions and comments were taken in consideration for face validity [4] and content validity. Face validity relates to a researcher's subjective judgement of whether the items in an instrument appear to be relevant, close, and reasonable, whereas content validity [5] is used to examine the accuracy of the domain being assessed.

**Fig. 1:**
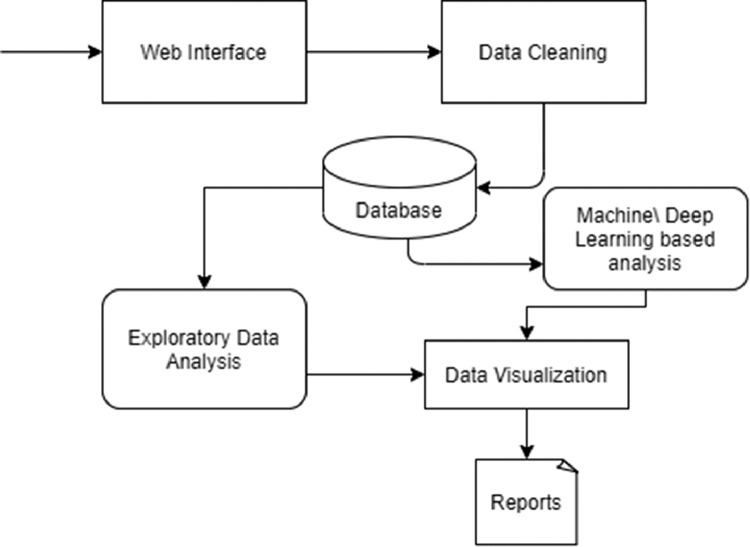
Proposed architecture for data analysis.

Accuracy can also be measured with different statistical tools like SPSS, ANOVA [6] etc. We have also built an architecture (Fig 1- Data Analysis) based on the datasets using machine learning models to generate Data visualization and final reports. In our datasets the demographic and socio-economic data were collected combinedly with a standard risk assessment tool HCR-20 which was customised as per the requirements of the research. Previous databases consist of either demographic profile or personality traits of an individual to do the behavior assessment [7] individually. Further we can use supervised machine learning approach to calculate the risk of reoffending among FTO's.

#### 2.1.1 Data quality control

To ensure the quality of each form was verified by the panel of expertise thoroughly and data were collected multiple times such that there won't be any biasness within the process. To avoid biasness the survey questionnaire information was filled four times in the span of 3 months.

#### 2.1.2 Exclusion criteria

All the participants information of survey questionnaire requires validation, which can only be done by the field expertise. The panel consists of different field expertise which are required for the exclusion of the features that are less prominent or useful for the current study. The insignificant attributes were listed in the Table 4 below. As the expertise have assigned the scores for each and every parameter an excluded some of the insignificant parameters which has less impact for the study.

**Table 4:** List of insignificant features.

Factors	Insignificant features p-value >0.05	p- value
Character, Financial and Socio-demographic Factors	Family Literacy*	0.0735
	Family sickness*	0.0672
	Family hierarchy*	0.0849
	House opinion *	0.0994
	Birthplace*	0.0759
	House relations*	0.0864
	Companion donations*	0.0649
	Emotional Violence*	0.0759

#### 2.1.3 Research design

A semi- structure proforma survey questionnaire was designed to collect information from each participant who were convicted once (FTO) in the state of Jharkhand India, currently 2 percent of the whole population of India are serving prison [8]. Jharkhand ranked 16th among 32 states of the country.

#### 2.1.4 Sample and location of study

A sample is a smaller set of items that is selected to represent the characteristics of a larger population. The current study's sample size could be increased to provide more reliable results. Random sampling procedures are used in the dataset of all 204 individuals to reduce bias. The level of significance were determined based on the p-value (where p-value is 0.05), standard statistical analysis were done to find the significant and insignificant features from the dataset. Anova test was employed to find the most significant features, i.e., tabulated in Table 5.

**Table 5:** Most Significant features in the data-set using Anova.

Sl.no	Most Significant Features	p-value
1.	Employment	0.0103
2.	Personality	0.0016
3.	Social Attitude	0.0054
4.	Self-Description	0.0095
5.	Social Belonginess	0.0025
6.	Offence	0.0055
7.	Parent Crime	0.0031
8.	H1- Previous Violence	0.0030
9.	H2- Other ASPD	0.0047
10.	H7- Personality disorder	0.0096
11.	H9- Violent attitudes	0.0017
12.	R2- Living Situation	0.0026
13.	C3 - Symptoms of Major Mental Disorder	0.0045

As seen in the table, these characteristics have a correlation with re-offending. The location of the study is the eastern part of India Jharkhand which contributes around 2 percent of the total convicted criminals of India [9].

#### 2.1.5 Procedure

The data of the questionnaire were taken and filled by the clinical psychologists with the consent of each individual participated. Each individual was informed that their data collected in the questionnaire are fully confidential. Every question was briefly explained to the participants such that he can answer appropriately. To fill the complete form for each participant it takes around 30-35 minutes

#### 2.1.6 Data analysis

The descriptive analysis of the expert panel was used to conduct the data analysis. The analysis was carried out using standard statistical methods

## Ethics Statement

The current study was conducted out in compliance with the Declaration, and all subjects gave their informed permission. As previously stated, the study was approved by both the Ethics Committee of the relevant institution, 10.13039/501100006464Birla Institute of Technology, Ranchi, India No. CSE/PHD/2017/09.

## CRediT Author Statement

**Aman Singh:** Methodology, Writing and Editing – Original draft; **Subrajeet Mohapatra:** Guidance, Approach, Corrections, Editing and review; **Madhumita Bhattacharya:** Conceptual Validation, Pilot data Scoring.

## Declaration of Competing Interest

According to the researchers, they will have no significant competing interests or personal financial concerns that could have distorted the result of this research.

## Data Availability

First Time Offender (Original data) (Mendeley Data).

## References

[1] Singh A., Mohapatra S. (2021). Development of risk assessment framework for first time offenders using ensemble learning. IEEE Access.

[2] Douglas K.S., Webster C.D. (1999). The HCR-20 violence risk assessment scheme: concurrent validity in a sample of incarcerated offenders. Crim. Just. Behav..

[3] Wilkes N., Anderson V.R., Johnson C.L., Bedell L.M. (2021). Mixed methods research in criminology and criminal justice: a systematic review. Am. J. Crim. Just..

[4] Oluwatayo J.A. (2012). Validity and reliability issues in educational research. J. Educ. Soc. Res..

[5] Anastasi A., Urbina S. (1997).

[6] Cardinal R.N., Aitken M.R.F. (2013).

[7] Ellis H. (1991).

[8] Govt.O.H.A.I. National Crime Bureau (2021).

[9] Hazra D. (2020). What does (and does not) affect crime in India?. Int. J. Soc. Econ..

